# Structural Changes in Milled Wood Lignin (MWL) of Chinese Quince (*Chaenomeles sinensis*) Fruit Subjected to Subcritical Water Treatment

**DOI:** 10.3390/molecules26020398

**Published:** 2021-01-13

**Authors:** Wen-Yue Wang, Zhao Qin, Hua-Min Liu, Xue-De Wang, Jing-Hao Gao, Guang-Yong Qin

**Affiliations:** 1 School of Life Sciences, Zhengzhou University, Zhengzhou 450001, China; 15238646131@163.com; 2 College of Food Science and Technology, Henan University of Technology, Zhengzhou 450001, China; qinzhao505@163.com (Z.Q.); wangxuede1962@126.com (X.-D.W.); g123asd321@163.com (J.-H.G.)

**Keywords:** Chinese quince, subcritical water, MWL, structure

## Abstract

Subcritical water treatment has received considerable attention due to its cost effectiveness and environmentally friendly properties. In this investigation, Chinese quince fruits were submitted to subcritical water treatment (130, 150, and 170 °C), and the influence of treatments on the structure of milled wood lignin (MWL) was evaluated. Structural properties of these lignin samples (UL, L130, L150, and L170) were investigated by high-performance anion exchange chromatography (HPAEC), FT-IR, gel permeation chromatography (GPC), TGA, pyrolysis-gas chromatography/mass spectrometry (Py-GC/MS), 2D-Heteronculear Single Quantum Coherence (HSQC) -NMR, and ^31^P-NMR. The carbohydrate analysis showed that xylose in the samples increased significantly with higher temperature, and according to molecular weight and thermal analysis, the MWLs of the pretreated residues have higher thermal stability with increased molecular weight. The spectra of 2D-NMR and ^31^P-NMR demonstrated that the chemical linkages in the MWLs were mainly β-*O*-4′ ether bonds, β-5′ and β-β′, and the units were principally G- S- H- type with small amounts of ferulic acids; these results are consistent with the results of Py-GC/MS analysis. It is believed that understanding the structural changes in MWL caused by subcritical water treatment will contribute to understanding the mechanism of subcritical water extraction, which in turn will provide a theoretical basis for developing the technology of subcritical water extraction.

## 1. Introduction

Chinese quince (*Chaenomeles sinensis*; Rosaceae) is native to China and is now widely planted in China, Korea, and Japan. The fruits of the Chinese quince are seldom eaten raw due to their hard, gritty, astringent flesh; usually they are processed into quince wine, starch syrup, quince tea, and sweets [1]. In recent years, components extracted from Chinese quince fruits, such as polyphenols, flavonoids, and organic acids, have been found to have considerable antioxidant and anti-inflammatory properties [2,3]. However, Chinese quince fruit lignin, although abundant, is not being used and has been rarely studied, especially in terms of its structural features [4].

The lignin in Chinese quince fruit cell wall, as a network of polymers, primarily consists of guaiacyl (G), sinapyl (S), and *p*-hydroxyphenyl (H) units, and these three units are linked by aryl ether and carbon–carbon bonds [5]. In addition, there is a good deal of evidence of lignin–carbohydrate complexes (LCC) [6]. The complexity and lack of homogeneity of Chinese quince fruit components means that they are difficult to separate, which strongly restricts their applications. Therefore, a more efficient technology for separating components from the cell wall of the fruits is an important prerequisite for developing the structural components of the fruits into valuable products [7].

Up to now, various technologies have been successfully developed and applied to isolate structural components of lignocellulose material. These technologies can be categorized into chemical (alkaline extraction, acid extraction), physical (steam explosion, subcritical water separation), and biological methods (enzymatic hydrolysis) [8,9,10]. Among these leading technologies, subcritical water extraction has attracted attention in recent years because it is considered more environmentally friendly [10]. In subcritical water treatment, the water present in the raw material fulfills every possible role in pretreatment as solvent, acid/base catalyst precursor, and reactant [11]. Thus, it does not require toxic or expensive solvents, and it eliminates the typically high energy cost of drying wet materials [12]. There are numerous findings describing the separation of lignin from wood or pulp with the purpose of demonstrating structural changes during subcritical water treatment [13]. Subcritical water extraction for lignin separation has been investigated using wheat straw, corn stover [14], poplar [15], eucalyptus [16], and bamboo [17]. Compared to these materials, Chinese quince has the advantages of huge production, low cost, and wide distribution; it is a plant of great value that has not been fully exploited.

It is reported that lignin from different sources and processes has distinct characteristics that may be related to the recalcitrance of the pretreated substrates [18]. As far as we know, no studies have been published on the structural characteristics of lignin extracted from Chinese quince fruits by subcritical water treatment. Understanding these structural properties is essential for developing industrial applications of Chinese quince [17]. Furthermore, understanding how subcritical water treatment alters Chinese quince lignin will contribute to understanding the general mechanism of subcritical water extraction, which in turn will provide a theoretical basis for developing the technology of subcritical water extraction.

Therefore, the purpose of this study was to investigate the structural changes in lignin extracted from Chinese quince fruits subjected to subcritical water treatment. Milled wood lignin (MWL) was isolated from untreated and treated Chinese quince fruits [1,5]. The influences on the structural features of MWL by the subcritical water pretreatment were evaluated via high-performance anion exchange chromatography (HPAEC), FT-IR spectroscopy, gel permeation chromatography (GPC), thermogravimetric analysis (TGA), pyrolysis-GC/MS analysis, and 2D-HSQC-NMR, as well as ^31^P-NMR.

## 2. Results and Discussion

### 2.1. Chemical and Morphological Changes of Chinese Quince Fruit Residues during Subcritical Water Treatment

**Figure 1:**
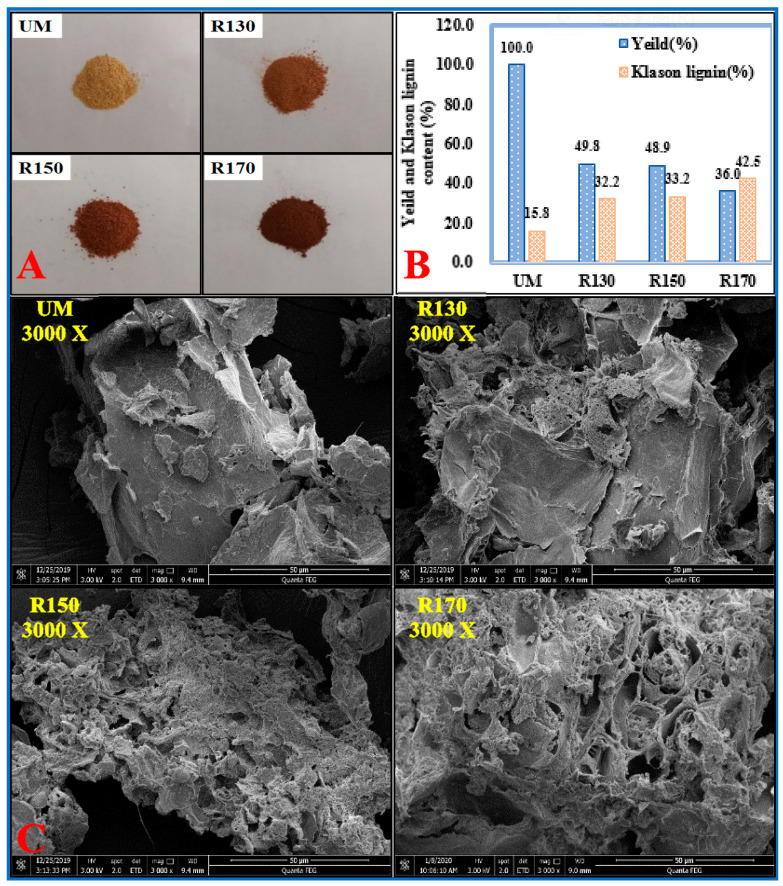
(**A**) Morphological changes in Chinese quince fruits residues before and after subcritical water treatment. (**B**) Yield (% dry initial material) and Klason lignin content of the Chinese quince fruit residues before and after subcritical water pretreatment. (**C**) SEM images of samples obtained from Chinese quince fruit residues before and after subcritical water treatment. UM, R130, R150, and R170 represent the untreated and pretreated Chinese quince by subcritical water at 130, 150, and 170 °C, respectively.

Changes in the surface morphology and Klason lignin content of Chinese quince fruits residues caused by subcritical water treatment at different temperatures are shown in Figure 1. The SEM photos reveal the details of ultra-structural alteration in the pretreated residues. Compared with the relatively tight and smooth surfaces of the untreated Chinese quince pulp residue samples, the surfaces of the treated residues were rougher and more severely disordered, and the color deepened with the increase of pretreatment temperature [19]. Irregularly shrunken, porous structures on the surfaces of samples became more and more prominent as the severity of the subcritical water treatment increased. This may be the consequence of changes in cell wall components during the subcritical water process under high heat [14,17].

Furthermore, it can be found that the yield of the solid residue decreased gradually from 49.8% to 36.0% as the temperature of subcritical water treatment ascended from 130 to 170 °C, indicating that the yield of solids was directly affected by pretreatment temperature. It should be noted that the yield of Chinese quince fruit residue was clearly far below previous investigations of wood after subcritical water treatment [20]. In particular, the material pretreated at 170 °C yielded only 36% residue. This low yield may be because Chinese quince fruit is particularly rich in both pectin and polysaccharides (11% of dry weight), which easily break down at very high temperatures [21]. In contrast with solid residue, the content of Klason lignin increased from 15.8% to 42.5%, as the subcritical water treatment temperature increased from 130 to 170 °C. These figures indicate that heating mostly eliminated the cell wall polysaccharides, thereby concentrating lignin [22].

### 2.2. Yield and Carbohydrate Components of MWLs

Many studies have found that the distribution and structural features of lignin in biomass are greatly affected by subcritical water treatment [14,17]. In particular, subcritical water treatment appears to localize and concentrate lignin in the cell walls; thus, in the subsequent extraction, MWL was isolated more easily [16]. Meanwhile, this temperature-related migration and localization explains why the MWL yields rose from 0.04% to 3.33% as the temperature of treatment increased. To determine the purity of the extracted MWL samples, the composition of the associated carbohydrate was determined, and the results are displayed in Table 1. It can be seen that low amounts of carbohydrates remained in all MWL fractions, even though these fractions had been purified. The fact that galacturonic acid, the main structural element of pectin, was the major monosaccharide in the UL samples indicates that the associated carbohydrates in the ULs originated from pectins. However, xylan is the main carbohydrate in the MWL isolated from other materials, such as bamboo, wood, and grass. Galacturonic acid in the MWL samples degrades rapidly as the temperature of the treatment rises. A previous publication has suggested that the reduction of galacturonic acid content might be because the oligomers undergo hydrolysis into monomers and/or other forms of degradation [23], and this was confirmed by DeMartini et al. using a new glycome profiling technique [24]. Glycome profiling results demonstrate that subcritical water treatment leads to an initial observable loss of arabinogalactan and pectic epitopes as well as destruction of lignin–polysaccharide (lignin–pectin/arabinogalactan) linkage interactions [18]. Xu et al. further proposed that subcritical water treatment releases pectic polysaccharides [25]. Besides arabinose, galacturonic acid, and glucose, xylose represented a high percentage of total sugar. Clearly, the proportion of xylose increased significantly when the temperature rose to 170 °C, and it became the main monosaccharide in the L170 samples, indicating that the associated carbohydrates in the L170s originated from xylan. It is possible that the increase in the amount of xylose was caused by the degradation of other sugars [12]. Furthermore, xylan is made up of two fractions: susceptible and non-susceptible to hydrothermal processing. The susceptible xylan fraction was hydrolyzed to xylooligomers, which can be further decomposed into xylose and promote dehydration of xylose to furfural, while much of the non-susceptible xylan remained in the lignins [26].

During subcritical water treatment, the predominant reactions are depolymerization, which is caused by cleavage of β-*O*-4′ linkages and ester bonds, and repolymerization, which is due to acid-catalyzed condensation. In some dilute acid pretreatment processes, dehydration and repolymerization of polysaccharides (or polysaccharide degradation products) and lignin lead to the formation of a compound that is similar to lignin, termed pseudo-lignin. According to published literature, the concentration of H^+^ and OH^−^ in the water that underwent the subcritical treatment is no less than that in a weak acid or a weak base [27]. Furthermore, the recondensation reactions in subcritical water treatment under the harsh conditions were reflected in the results of GPC analysis.

### 2.3. FT-IR Analysis

**Figure 2:**
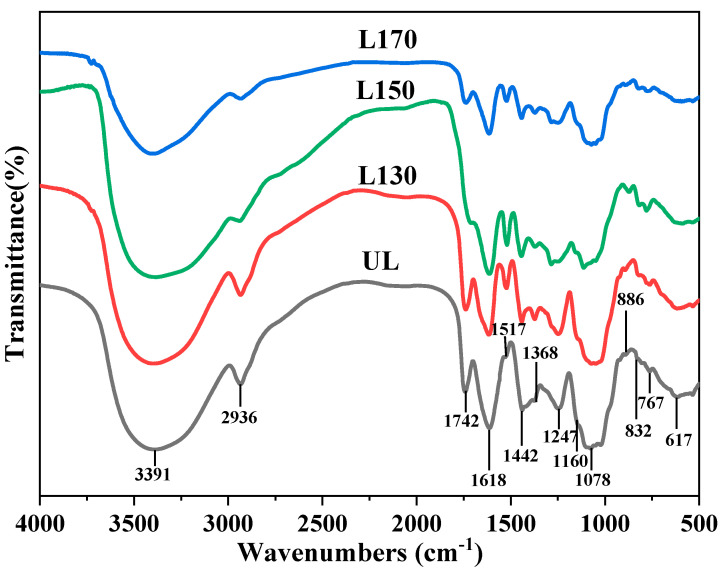
FT-IR spectra of UL, L130, L150, and L170 fractions.

For the purpose of observing the variation in the functional groups after subcritical water treatment, FT-IR spectra (Figure 2) of the four MWLs (UL, L130, L150, and L170) were obtained, and peaks were identified on the basis of published literature [28]. It can be seen that the basic structures of the lignin in the four samples are very similar. As shown, the peak at 3391 cm^−1^ exhibited a wide absorption band, resulting from the stretching vibration of O–H in aromatic and aliphatic groups [29], and the peak at 2936 cm^−1^ exhibited a narrow but prominent band, representing C–H stretching vibration, the band diminished in L150 and L170. The absorption at 1742 cm^−1^ originated from the C=O stretching in unconjugated ketones and carbonyl groups [30], the absorption band significantly diminished in L150 compared with others. This is in line with the carbohydrate content, GPC and the TGA data. The results suggested that L150 is the optimum pretreatment condition to obtain MWL. Peaks of typical aromatic structure vibrations and deformation of C–H in the lignin appear clearly at 1618 and 1517 cm^−1^, as well as at 1442 cm^−1^ [28]. The peaks at 1618, 1517, and 1442 cm^−1^ can be assigned to the characteristic absorption of benzene rings. Although their intensities differed, the presence of the bands confirmed that the “core” structure of lignin did not change dramatically during the subcritical water treatment [31]. Stretch signals occur at 1368 cm^−1^, which is the phenolic OH region of lignin. Signals at 1247 cm^−1^ are assigned to guaiacyl ring breathing with C=O stretching. The additional characteristic band of G- S- H- lignin at 1160 cm^−1^ shows that the isolated MWLs from the Chinese quince fruits belong to G- S- H- lignin according to the classification system of FT-IR suggested by Faix et al. [32]. The band at 1160 cm^−1^ implies an antisymmetric C–O stretching of ester groups. The bands at 832 and 1078 cm^−1^ are attributed to C–H out-of-plane deformation in 2, 5, and 6 positions and to C–H in-plane deformation stretching of G units, respectively. Finally, the absorption at 886 cm^−1^ is due to C–H deformation stretching of glucose rings [33].

### 2.4. Molecular Weight Distributions of MWLs

The question as to whether the depolymerization of lignin took place during the subcritical water treatment was answered by studying the molecular weight and its distribution for all MWL samples. Generally, recondensation and depolymerization are concerted reactions happening during subcritical water treatment [34]. Actually, the molecular weight of MWLs decreased when the bonds of β-*O*-4′ linkages were disrupted, and condensation reactions generally resulted in the increase of the lignin molecular size, along with the formation of heterogeneous and condensed structures. Table 2 depicts the changes in the weight-average (*M*_w_) and number-average (*M*_n_) molecular weight and the polydispersity (*M*_w_/*M*_n_) of the MWLs extracted from untreated and treated Chinese quince fruits. An obvious decline of the molecular weight of subcritical-water-treated MWL was observed in comparison with that of untreated MWL. The *M*_w_ of the lignin fraction UL was slightly higher than those of the other MWL preparations (L130, L150, and L170), which confirmed that more ether linkages of the MWLs were cleaved during subcritical water treatment [10,14]. However, the *M*_w_ and *M*_n_ of the preparations of samples L130 and L150 were slightly less than those of samples L170, which might be because the recondensation reaction at 170 °C was more severe than that at 130 and 150 °C. In general, more depolymerization than repolymerization occurred in the lignins under the subcritical water treatment. All the values of polydispersity of the pretreated MWLs were reduced, indicating that the molecular weight distribution was narrow and that the lignin fractions were homogenous macromolecules [16]. Polydispersity is another vital parameter in the field of natural macromolecule applications because narrow polydispersity indicates excellent physicochemical stability [31]. Thus, the lower molecular weight and narrow polydispersity of these samples make them appropriate for industrial applications.

### 2.5. Thermal Analysis

**Figure 3:**
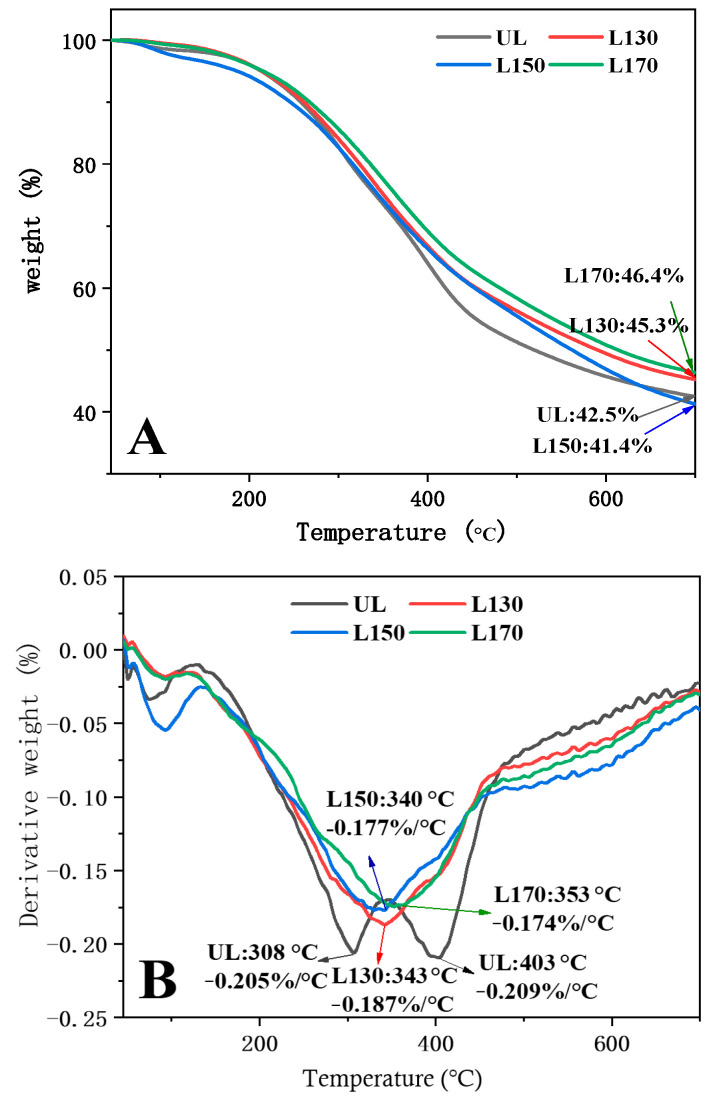
TG curves (**A**) and DTG (**B**) curves for UL, L130, L150, and L170 fractions.

In order to investigate the correlation between thermal properties and structure, the TGA and DTG of four MWLs were compared. The TGA and DTG curves of the four MWLs (UL, L130, L150, and L170) are shown in Figure 3. The TGA curves display the mass loss of volatile content and the mass of residual content. The temperatures at which the samples demonstrated the maximum speed of degradation are consistent with the peaks of the DTG curves. Normally for lignin, decomposition occurs over a wide range of temperature, with the range of 200 to 700 °C generally regarded as the primary degradation region. As shown in Figure 3A, for the four MWL fractions, the integrated process of pyrolysis could be categorized into three stages, as reflected in the TGA curves. At the first stage, when the temperature was below 160 °C, the volatilization of gases with lower molecular weight and of free water generated the weight loss. At the second stage, a relatively wide range of temperature from 200 to 500 °C, degradation was dramatic, with major loss of mass of the lignin fractions. During the last stage, when the temperature was above 500 °C, the reactions of decomposition and condensation in the aromatic rings of lignin samples occurred simultaneously.

As can be seen from the Figure 3A, a significant amount of residual char was obtained at 700 °C. More specifically, there were still 42.5%, 45.3%, 41.4%, and 46.4% of solid residues left for lignin fractions UL, L130, L150, and L170, respectively. To some extent, the residue content represents thermostability. These solid residues corresponded well with the molecular weights of the lignin fractions displayed in Table 2, revealing that thermostability improved with the increase of molecular weight in the pretreated samples. DTG_max_ appeared at 343 °C for the L130 sample, 340 °C for the L150 sample, and 353 °C for the L170 sample as seen in Figure 3B, while two peaks of DTG_max_ were showed at 308 and 403 °C in the UL. The first DTG_max_ perhaps relates to the pyrolysis of carbohydrates remaining in the UL preparations, while the second DTG_max_ was possibly in connection with the decomposition of lignin [33,35,36,37,38].

### 2.6. Py-GC/MS Analysis

Pyrolysis-gas chromatography/mass spectrometry (Py-GC/MS) is a sensitive and useful technique for the in situ characterization of plant constituents. The compositions of the four MWLs at the different pretreatment temperatures were examined in situ by Py-GC/MS [39]. During pyrolysis, the primary breakdown of lignin polymers occurred at the specific structural sites with low chemical-bond energy. The stable and volatile degradation fractions generated were split and analyzed by GC/MS, which provided valuable data about the structure of MWL polymers [40]. Their chromatograms are displayed in Supplementary Figure S1, and the main relative abundances and identities of the released compounds as well as structural formula of the lignin samples are shown in Supplementary Table S1 and Supplementary Figure S2, respectively.

The detected pyrolysis products of the MWLs fractions were categorized into three types on the basis of their structure, namely, guaiacyl (G), syringyl (S), and *p*- hydroxyphenyl (H) lignin units. Compared with the S-type phenols, G-type phenols were more abundant. S- or G-type phenolic monomers were obtained from monomeric units of MWL after cleavage of α- or β-aryl ether linkages. H-type phenols were produced from further degradation of the S- or G-type phenolic compounds [41]. As shown in Supplementary Figure S1, phenol, 2-methoxyphenol **(3)**, creosol **(5)**, catechol **(6)**, 2-methoxy-4-vinylphenol **(8)**, and 2,6-dimethoxy phenol **(10)** were identified as the main pyrolysis products. The lignin pyrolysis fragments with longer side chains, such as 2,4-bis(1,1-dimethylethyl)-phenol, 1-(2,6-dihydroxy-4-methoxyphenyl)-ethanone, 3,5-dimethoxy-4-homovanillyl alcohol, hydroxyphenylacetic acid, and 3,5-di-tert-butyl-4-hydroxyphenylpropionic acid, were obtained only from the MWL without subcritical water pretreatment. This may be because the lignin fragments were cracked by the subcritical water pretreatment, resulting in the production of compounds with fewer side chains.

The 2-methoxy-4-vinylphenol, found in considerably high content in the pyrolysis products of the MWLs, may be derived from ferulic acid and *p*-coumaric acid resulting from the acetylation of MWL side chains by decarboxylation during pyrolysis. Therefore, the 2-methoxy-4-vinylphenol **(8)** produced during the Py-GC/MS process cannot be included in calculations of the composition of H:G:S in the lignin because the main fragments are not part of lignin’s core structural units. Rough calculation of the S/G ratios (by ignoring the 2-methoxy-4-vinylphenol) gave 0.34–0.51 for the MWLs, suggesting that guaiacyl-rich oligomers took precedence during the extraction of MWL fragments [42]. However, it is noted that the S/G ratio increased slightly after subcritical water pretreatment, and the results are consistent with previous study [43]. These observations may be due to the degradation of β-*O*-4′ linkages, and consequently, the increased severity caused a release of more S-units. In contrast, the G units generally were more compacted because aromatic C5 were available for additional ether interunit or carbon–carbon bonds, making them relatively more resistant to lignin depolymerization during pretreatment under mild conditions. However, the degradation of G units occurred accompanied by severe conditions, which was well explained why the S/G ratio of the L170 sample was lower than that of the L130 and L150 samples.

### 2.7. NMR Analysis

#### 2.7.1. 2D-HSQC-NMR

**Figure 4:**
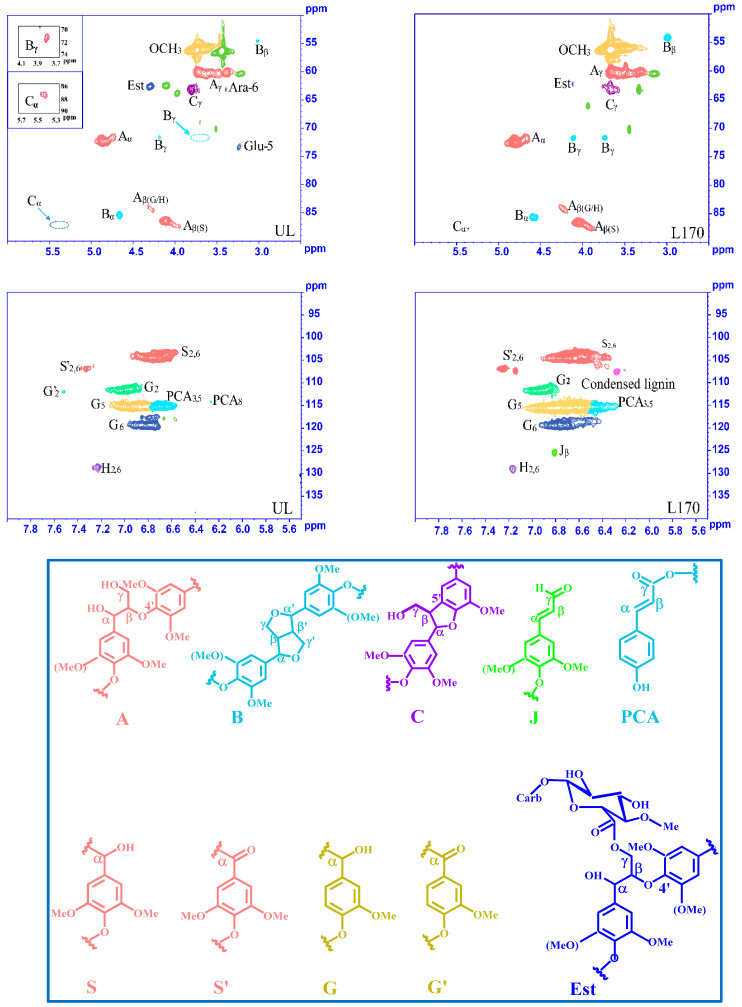
2D-HSQC-NMR spectra of UL and L170 fractions and the main lignin substructure linkages present in lignin fractions: (A) β-*O*-4 aryl ether linkages; (B) resinol substructures; (C) phenylcoumaran substructures; (J) cinnamaldehyde end groups; (PCA) *p*-coumaric acid (ester); (S) syringyl units; (S′) oxidized (C_α_=O) syringyl units; (G) guaiacyl units; (G′) oxidized guaiacyl units with a Cα ketone; (Est) γ-ester.

The technique of 2D-HSQC-NMR has great value for understanding the typical structural properties of lignin and changes in lignin during subcritical water treatment. In the present investigation, the structural properties of lignin polymers of two MWLs (UL and L170) were explored using 2D-HSQC-NMR. The side chain and aromatic regions of the 2D HSQC-NMR spectra of UL and L170 and the principal substructures of MWLs are exhibited in Figure 4; the signals were assigned by comparing with the published literature and are listed in Supplementary Table S2.

In the side-chain region (δ_C_/δ_H_ 50–90/2.5–6.0 ppm) of the spectra, the cross-signals of various interunit linkages and substructures of the lignin fractions, such as β-aryl ethers (β-*O*-4′, A), resinols (β-β′, B), and phenylcoumarins (β-5′, C) were identified. As can be seen from Figure 4, the signals of methoxyl groups (OCH_3_, δ_C_/δ_H_ 55.4/3.68 ppm) and β-*O*-4′ aryl ether bonds were clear [44]. The C_α_–H_α_ correlations in β-*O*-4′ substructures were seen at δ_C_/δ_H_ 72.4/4.81 ppm, whereas the correlations of C_β_–H_β_ were found at δ_C_/δ_H_ 84.3/4.23 and 86.7/4.06 ppm for substructures connected to G and S units, respectively. The signals at δ_C_/δ_H_ 60.4/3.30 to 3.79 ppm reflected the correlation of C_γ_–H_γ_ in β-*O*-4′ substructures and were partially overlapped by other signals. The presence of resinol (β-β′, B) was proved by C–H correlations at δ_C_/δ_H_ 85.7/4.59, 54.3/3.00, and 71.8/ (3.75, 4.12) ppm for C_α_/H_α_, C_β_/H_β_, and C_γ_/H_γ_, respectively. Signals correlating to phenylcoumaran (β-5′, C) substructures were observed to be fairly weak in the spectra. The signals correlating to C_α_–H_α_ appear at δ_C_/δ_H_ 87.7/5.38 ppm, and the C_γ_–H_γ_ correlations are seen at δ_C_/δ_H_ 63.3/3.66 ppm [45,46]. Interestingly, UL showed the C_6_–H_6_ correlation of arabinosyl units Ara-6 at 63.3/3.40 ppm and C_5_–H_5_ correlations in glucosyl units Glu-5 at 73.3/3.25 ppm, respectively, but neither of these were detected in the HSQC spectra of L170. This suggests that the polysaccharides associated with lignin were dissolved and degraded during subcritical water treatment [44]. Furthermore, it is worth noting that the γ-ester structure, which exists in the lignin–carbohydrate complex (LCC), occurred in both UL and L170, meaning that the structure of γ-ester tends to stay stable during the integrated treatment [47]. The cross-signals of guaiacyl (G), syringyl (S), and *p*-hydroxyphenyl (H) units were clearly recognized according to the corresponding correlations of those substructures at the regions (δ_C_/δ_H_ 90–140/5.5–8.0 ppm) of aromatics in the MWL spectra. Obviously, the S-lignin units exhibited signals for the C_2,6_–H_2,6_ correlation at δ_C_/δ_H_ 104.5/6.63 ppm, and the C_2,6_–H_2,6_ correlations in C_α_-oxidized S units (δ_C_/δ_H_ 107.0/7.26 ppm) were also present in the MWL spectra. What is noteworthy is that the signals of condensed S units appeared at δ_C_/δ_H_ 107.7/6.27 ppm in the L170 spectra but were absent in the spectrum of UL. The cross-signals at δ_C_/δ_H_ 111.8/6.94, 115.6/6.65, and 119.6/6.75 ppm belong to the C_2_–H_2_, C_5_–H_5_, and C_6_–H_6_ of the G units, respectively. A small fraction of oxidized G units with a C_α_ ketone (G′) exhibited correlation for C_2_–H_2_ (δ_C_/δ_H_ 112.0/7.53 ppm) in the UL spectra, but no correlated signals of G′ occurred in the corresponding regions of the L170 spectra [32,46]. The related signal of C_2,6_–H_2,6_ in the H-lignin units was identified at δ_C_/δ_H_ 129.2/7.17 ppm. Other cross-signals appeared and were assigned to *p*-coumaric acid (PCA) and cinnamaldehyde end groups (J). In this research, the main signals related to PCA substructures were discovered in the spectra of both UL and L170. The correlation signals of PCA were observed relatively strongly at δ_C_/δ_H_ 115.2/6.71 ppm (PCA_3,5_) and 114.2/6.27 ppm (PCA_8_). The clear signals at δ_C_/δ_H_ 125.97/6.74 ppm were attributed to C_β_–H_β_ correlation in cinnamaldehyde end groups (J) [48].

The technique of 2D-HSQC-NMR provided proof of the precise structural evolution in MWL fragments after subcritical water treatment. In the 2D-HSQC spectra, information about the primary bonds in the lignin polymers and their relative abundances were obtained. The relative percentages of each linkage bond (β-*O*-4′, β-β′, β-5′, and S/G ratios) are displayed in Table 3. As expected, the linkages of β-*O*-4′ proved to be the most abundant in the MWL, which accounted for up to 72.26–74.11% of all interunit linkages, followed by resinols (β-β′) and the phenylcoumarans (β-5′) with lower contents [1]. The portion of β-*O*-4′ linkages in L170 was slightly lower than that of UL, illustrating that depolymerization occurred during subcritical water treatment, which resulted in fractured linkages of β-*O*-4′ [34]. Depolymerization and repolymerization occurred concurrently during the subcritical water treatment. As shown in the spectra, the signals from the condensed S units appeared in the 2D-HSQC spectra of L170, implying that the condensation reaction occurred in the MWL during the subcritical water treatment. In addition to the broken β-*O*-4′ linkages and the variation of carbon–carbon linkage, it should be noted that the changes of the S/G ratio were also mainly due to structural variation observed after subcritical water treatment [16]. The S/G ratios were calculated roughly to be 1.48 and 3.37 for UL and L170, respectively. Clearly, the S/G ratio increased after subcritical water treatment, which implied that more S-units were released with the degradation of β-*O*-4′ linkages during the treatment.

#### 2.7.2. ^31^P-NMR Analysis

^31^P-NMR is a valuable technique for quantifying various hydroxyl groups in lignin. For the purpose of learning more about the influence of subcritical water treatment on the structure of functional groups in lignin, four MWL polymers were phosphitylated and compared using quantitative ^31^P-NMR. As is shown from Table 4, the concentration of aliphatic OH, phenolic OH, guaiacyl OH, *p*-hydroxyphenyl OH, and carboxylic OH (in mmol/g) were determined. The ^31^P-NMR analysis confirmed that the MWLs extracted from Chinese quince fruits were predominantly composed of G-, S-, and H-type units, which is consistent with the results of pyrolysis-GC/MS analysis. The aliphatic OH contents in the MWL samples that had been detected were not significantly different. The result is inconsistent with the previous study, which showed the decline of aliphatic OH contents accompanied a rise in temperature. The inconsistency may be due to a difference in conditions. Under the relatively mild conditions of this study, aliphatic OH modification rarely occurs [16]. Moreover, the contents of S–OH groups in all lignin fractions were lower than G–OH groups, indicating that majority of S-type lignin units participated in β-*O*-4′ linkages, while only a small quantity of S–OH reacted with TMDP and were thus recorded by the ^31^P-NMR [44]. Additionally, it is possible that linkages of the aryl ether cracked, resulting in increases of syringyl OH and guaiacyl OH as the treatment severity increased; this phenomenon has been reported in a previous publication [49,50]. These conclusions are consistent with the molecular weight revealed by GPC analysis. In addition, it was discovered that *p*-hydroxyphenyl OH contents increased from 0.05 to 0.14 mmol/g, suggesting that demethoxylation occurred at 3-position of G units or 3,5-position of S units under more severe treatment conditions [38]. It is worth noting that there was no obvious difference in the content of hydroxyl groups among UL, L130, and L150 samples; however, the numbers of phenolic hydroxyl groups and carboxylic groups in L170 increased significantly, implying greater reaction activity at the highest pretreatment temperature.

## 3. Materials and Methods

### 3.1. Materials

Fruits from mature Chinese quince plants were collected in Zhengzhou Orchid Engineering Technology Research Center, China. Seeds were removed, and the flesh was cut into 3–5 mm thick slices, and dried (50 °C, 24 h). The air-dried materials were milled in an electric grinder and sieved into 20–40 mesh fractions. The fractions were submitted to successive Soxhlet extraction with benzene/ethanol (2:1, *v*/*v*) for 12 h; the insoluble portions were air-dried. All standard chemicals were reagent or analytical grade.

### 3.2. Subcritical Water Treatment

Subcritical water extraction was performed in a 500 mL reactor (model SLM-500, Sen Long Instruments Company, Beijing, China), equipped with a modular electric heating system and a control system. In a typical subcritical water experiment, Chinese quince fruit powder and water were mixed in a solid to liquid ratio of 1:10 (g/mL), and put into the extractor. The pretreatment temperatures (130, 150, and 170 °C) were maintained for 10 min with an agitation speed of 500 rpm, and the pressure of the system was about 0.3, 0.5, and 0.8 MPa, respectively. After extraction, the reactor was immediately taken out of the heater and cooled by running tap water to stop the reaction. The solid residue was recovered by filtration, washed with deionized water until the solvent became colorless, and oven dried at 50 °C. The pretreated residues were labeled as R130, R150, and R170, corresponding to the pretreatment temperature. Meanwhile, a set of samples without subcritical water treatment were prepared and labeled as UM. The content of Klason lignin in samples (UM, R130, R150, and R170) was determined using the standard NREL (National Renewable Energy Laboratory) analytical method.

### 3.3. Scanning Electron Microscopy (SEM)

Each sample was placed onto a circular metal plate and then sputter-coated with gold. The microscopic characteristics of these samples were examined on a FEI Quanta 250 SEM (Thermo Fisher Scientific, Hillsboro, OR, USA) at 25 kV [51].

### 3.4. Preparation of MWL Fractions

Milled wood lignin (MWL) from fruits of Chinese quince was isolated according to a method described by Björkman [52]. The materials (UM, R130, R150, and R170) were treated by means of ball milling (XQM-2, Changsha Tianchuang Powder Technology Co., Ltd., Changsha, China) at 400 rpm, for 25 h with breaks of 5 min after each 20 min of milling. Dioxane-water (96:4, *v*/*v*) was used to extract the powder after ball-milling for 24 h at room temperature, using a solid-to-liquid ratio of 1:10 (g/mL); this procedure was repeated twice for a total of three times. The solution was centrifuged. The supernatant was collected and then evaporated at 45 °C under vacuum to dryness to obtain crude lignin. Deionized water was used to wash the crude lignin until the filtrate was clear. Next, the crude lignin was dissolved in 90% acetic acid, then precipitated with deionized water. The precipitated lignin was further purified according to the procedure reported by Björkman [52]. The four lignin fractions obtained from solid residue were designated UL, L130, L150, and L170.

### 3.5. Structural Characterization

The carbohydrate moieties related to MWLs were measured by dilute sulfuric acid hydrolysis. The samples of MWL, each weighing 4–6 mg, were hydrolyzed for 45 min with 0.125 mL of 72% H_2_SO_4_. Then, the solution of H_2_SO_4_ was diluted with 1.35 mL ultrapure water at 105 °C for 150 min. Moreover, the reaction system needed to be shaken every 30 min during the reaction. Subsequently, to filter out unhydrolyzed fractions, a 0.22 μm PTFE filter was used, and the filtrate was diluted 50-fold, then analyzed by Dionex ICS3000 HPAECwith an ion exchange Carbopac PA-1 column (4 × 250 mm) (Dionex Corporation, Sunnyvale, CA, USA) and a pulsed amperometric detector [53].

FT-IR analysis of MWL fractions was carried out on a type-iS10 FT-IR microscope (Thermo Nicolet Corporation, Madison, WI, USA) using a KBr disk containing 1% samples, which were ground finely. The spectra were collected within the range of 4000 to 400 cm^−1^ [6].

The molecular weights of the MWLs were measured with GPC on a PL-gel 10 mm Mixed-B 7.5 mm ID column, using monodispersed polystyrene for calibration. Acetylation was performed on all samples prior to determination. MWL samples weighing 100 mg were added into acetic anhydride/pyridine (4 mL) and stirred in the dark for 24 h. Next, 2 mg freeze-dried samples that had been acetylated were dissolved in 2 mL tetrahydrofuran and filtered; 10 μL filtrate was injected into an injection system. The flow rate was maintained at 1 mL/min, and the column was at room temperature [54].

Thermogravimetric (TGA) and differential thermogravimetric (DTG) analyses were used to evaluate the thermal properties of MWLs with a simultaneous thermal analyzer (NETZSCH STA 449C). The MWL samples weighing about 4–8 mg were heated to 700 °C in an atmosphere of nitrogen with a heating rate of 10 °C/min [47,55].

Pyrolysis-gas chromatography/mass spectrometric (Py-GC/MS) studies were conducted with a CDS5000 pyrolyzer equipped with an Agilent GC/MS system. The samples (500 μg) were firstly pyrolyzed at 500 °C for 20 s. The oven temperature was controlled as follows: First, the temperature was raised to 50 °C and maintained for 4 min. Subsequently, the temperature was raised to 100 °C at a rate of 10 °C/min and then maintained for 5 min. Finally, the temperature was increased to 280 °C at the rate of 6 °C/min [33].

A Bruker spectrometer TYPE-AVIII 400 MHz (Bruker Co., Swiss) gathered the 2D-NMR spectra of MWLs samples in DMSO-*d6* at 25 °C. The MWLs samples (25 mg) were dissolved in DMSO-*d6* (0.5 mL), a deuterium-generation reagent; the spectral width of ^1^H NMR and ^13^C-NMR were set at 5000 and 20,000 Hz, respectively [32,56]. ^31^P-NMR spectra were obtained after in situ derivatization of the MWLs with TMDP (2-chloro-4,4,5,5-tetramethyl-1,3,2-dioxaphospholate); cyclohexanol was used as internal standard. The parameters for ^31^P-NMR were as follows: 25 s pulse delay, 256 transients, and a 90° pulse angle [45].

## 4. Conclusions

In the present work, subcritical water treatment was used to treat Chinese quince fruits. The MWL polymers isolated from the pretreated residues were of very high purities. The conditions of subcritical water treatment broke the chemical linkages between lignin and hemicelluloses in the cell walls of Chinese quince fruit samples to some extent, and thus increased the yield of lignins. It was found that glucose and galacturonic acid were the predominant carbohydrates of these lignin samples. More remarkable, the amount of xylose in the MWLs of Chinese quince fruits after subcritical water treatment increased dramatically with rising temperature, which may be due to the degradation of other sugars. The GPC technique explained that the fragmentation of interunitary bonds in lignins (β-*O*-4′, β-β′, and β-5′) during subcritical water treatment took place with the reactions of repolymerization, simultaneously. Thermal analysis and NMR results confirmed that the MWLs were predominantly composed G-, S- and H-type units, and the conclusion consistent with the results of pyrolysis-GC/MS analysis. It is believed that understanding the structural variation of lignin under subcritical water treatment will contribute to understanding quince fruits cell wall structure, thereby leading to better applications of Chinese quince fruits and a more complete theoretical basis for using the technology of subcritical water extraction.

## Figures and Tables

**Table 1:** Yields and carbohydrate contents of lignin fractions.

Sample ^a^	Yield ^b^ (%)	Sugar Content (%)	Carbohydrate Composition Content (%)						
			Fuc ^c^	Ara ^c^	Gal ^c^	Glc ^c^	Xyl ^c^	Gal-A ^c^	Glc-A ^c^
UL	0.04	0.62	0.06	1.05	6.12	20.60	1.08	62.63	8.47
L130	0.39	0.16	0.68	3.88	10.70	23.08	11.42	47.92	2.34
L150	0.54	0.11	0.83	10.22	7.28	21.14	8.21	50.41	1.91
L170	3.33	0.25	1.14	19.70	3.63	8.98	44.61	21.61	0.33

^a^ UL, L130, L150, and L170 represent the milled wood lignin (MWL) extracted from solid residue (SR, R130, R150, and R170), respectively. ^b^ Based on solid residue (wt.%). ^c^ Abbreviation: Fuc, fructose; Ara, arabinose; Gal, galactose; Glc, glucose; Xyl, xylose; Gal-A, galacturonic acid; Glc-A, glucuronic acid.

**Table 2:** Weight-average (*M*_w_) and number-average (*M*_n_) molecular weights and the polydispersity (*M*_w_/*M*_n_) of UL, L130, L150, and L170 samples.

Sample	*M*_w_	*M*_n_	*M*_p_	*M*_w_/*M*_n_
UL	16,543	5276	16,190	3.14
L130	12,204	6086	7801	2.01
L150	10,516	5444	8389	1.93
L170	13,524	6648	10,807	2.03

**Table 3:** Quantification of the UL and L170 by quantitative 2D-HSQC-NMR method.

Lignin Interunit Linkages	Percentage (%)	
	UL	L170
β-*O*-4′ aryl ethers (A)	74.11	72.26
β- β′ (resinols) (B)	19.78	21.08
β-5′ (phenylcoumarans) (C)	6.11	6.66
Syringyl units (S_2,6_)	69.04	82.83
Guaiacyl units (G_2_)	23.34	12.29
*p*-Hydroxyphenyl units (H_2,6_)	7.63	4.88
S/G ratio	1.48	3.37

**Table 4:** Quantification of the UL, L130, L150, and L170 fractions by quantitative ^31^P-NMR analysis (mmol/g).

Samples	Aliphatic OH	Syringyl OH	Guaiacyl OH	*p*-Hydroxy Phenyl	Carboxylic Group
UL	1.30	0.04	0.36	0.05	0.02
L130	1.33	0.04	0.30	0.05	0.06
L150	0.92	0.04	0.24	0.06	0.06
L170	1.30	0.23	1.07	0.14	0.16

## Data Availability

The data presented in this study are available on request from the corresponding author.

## Supplementary Materials

Figure S1: Py-GC/MS chromatograms of UL, L130, L150, and L170 fractions. Figure S2: The structural compounds labeled in Table S1. Table S1: The identities and relative abundances of carbohydrate-derived compounds by Py-GC/MS. Table S2: Assignments of main lignin 13C–1H cross signals in the 2D HSQC spectra of the UL and L170.

## Sample Availability

Samples of the compounds are available from the authors.
