# Supplementary material for: Structural Changes in Milled Wood Lignin (MWL) of Chinese Quince (Chaenomeles sinensis) Fruit Subjected to Subcritical Water Treatment
Source: Molecules. 2021 Jan 13;26(2):398. doi: 10.3390/molecules26020398 (PMC7828612; doi:10.3390/molecules26020398)
Supplement: Supplementary file 1 [file molecules-26-00398-s001.pdf]

Supplementary Information

Structural changes in milled wood lignin (MWL) of Chinese quince (*Chaenomeles sinensis*) fruit subjected to subcritical water treatment

Wen-Yue Wang [1, 2], Zhao Qin [2], Hua-Min Liu [2,*], Xue-De Wang [2], Jing-Hao Gao [2], Guang-Yong Qin [1, *]

[1] School of Life Sciences, Zhengzhou University, Zhengzhou 450001, China; 15238646131@163.com

[2] College of Food Science and Technology, Henan University of Technology, Zhengzhou 450001, China; qinzhao505@163.com (Z.Q.); wangxuede1962@126.com (X.-D.W.); g123asd321@163.com (J.-H.G.)

* Correspondence: hmliu@haut.edu.cn (H.-M.L.); qinguangyong@zzu.edu.cn (G.-Y.Q.)

Contents

Figure. S1 Py-GC/MS chromatograms of UL, L130, L150, and L170 fractions.

Figure. S2 The structural compounds labelled in Table S1

Table S1 The identities and relative abundances of carbohydrate-derived compounds by Py-GC/MS.

Table S2 Assignments of main lignin $^{13}C–^{1}H$ cross signals in the 2D HSQC spectra of the UL and L170.

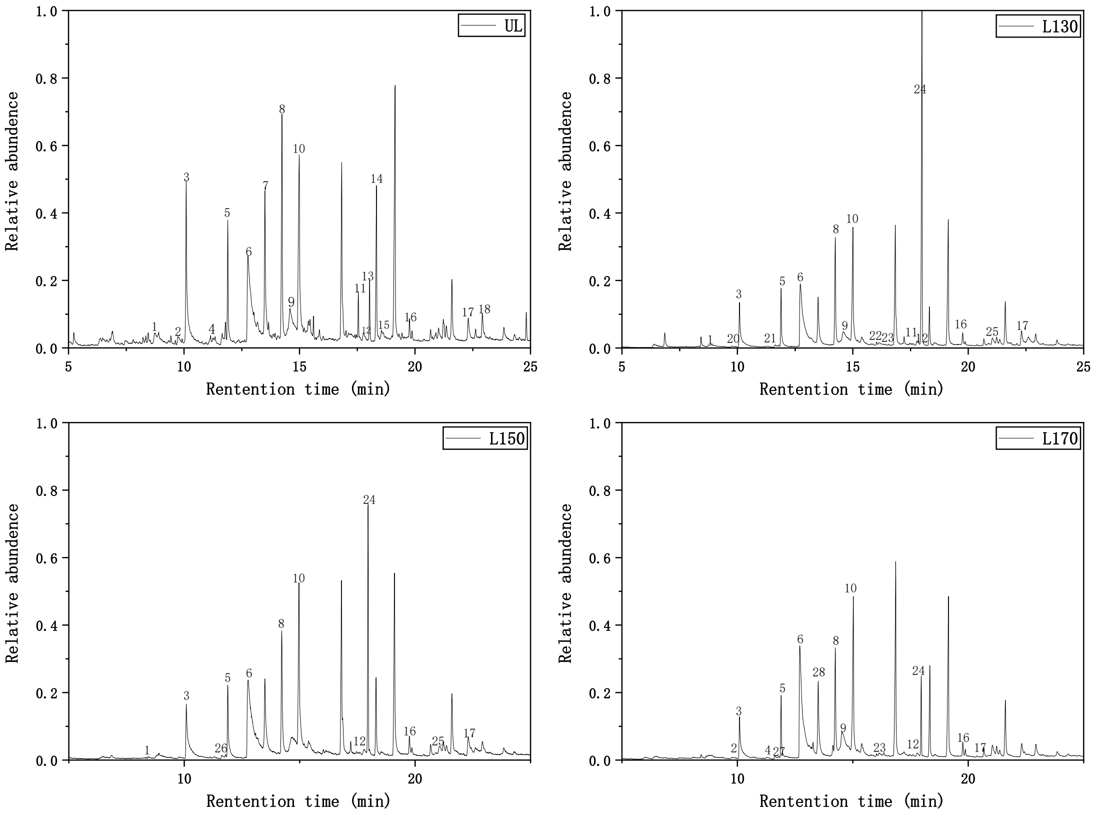

Figure S1. Py-GC/MS chromatograms of UL, L130, L150, and L170 fractions.

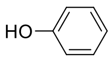
1
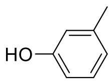
2
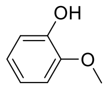
3
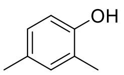
4
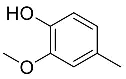
5
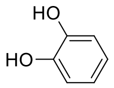
6
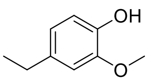
7
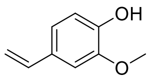
8
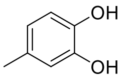
9
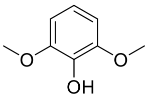
10
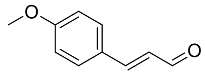
11
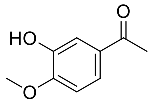
12
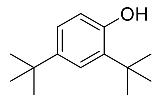
13
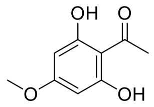
14
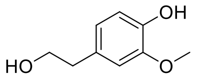
15
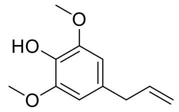
16
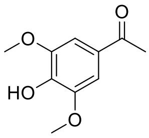
17
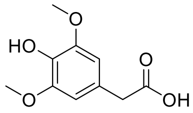
18
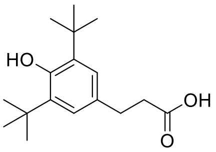
19
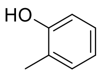
20
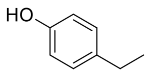
21
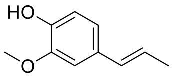
22
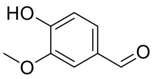
23
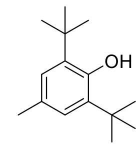
24
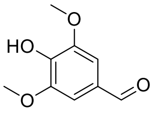
25
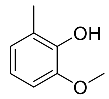
26
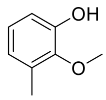
27
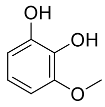
28

Figure S2. The structural compounds labelled in Table S1

Table S1

The identities and relative abundances of carbohydrate-derived compounds by Py-GC/MS.

NO.	Compound	Origin	UL	L130	L150	L170
1	Phenol	H	1.4	0.1	0.4	0.0
2	Phenol, 3-methyl-	H	0.5	0.0	0.0	0.2
3	Phenol, 2-methoxy-	G	12.7	7.8	11.4	7.7
4	Phenol, 2,4-dimethyl-	H	0.6	0.0	0.0	0.5
5	Creosol	G	6.1	5.7	7.1	5.5
6	Catechol	G	18.7	24.3	28.6	33.9
7	Phenol, 4-ethyl-2-methoxy-	G	9.9	0.0	0.0	0.0
8	2-Methoxy-4-vinylphenol	G	13.6	11.5	12.2	10.0
9	1,2-Benzenediol, 4-methyl-	G	3.0	2.2	0.0	4.2
10	Phenol, 2,6-dimethoxy-	S	15.1	13.8	18.3	16.5
11	trans-4-Methoxycinnamaldehyde	G	0.1	0.1	0.0	0.0
12	Ethanone, 1-(3-hydroxy-4-methoxyphenyl)-	G	0.9	0.8	0.5	0.5
13	Phenol, 2,4-bis(1,1-dimethylethyl)-	H	2.9	0.0	0.0	0.0
14	Ethanone, 1-(2,6-dihydroxy-4-methoxyphenyl)-	G	8.5	0.0	0.0	0.0
15	Homovanillyl alcohol	G	0.4	0.0	0.0	0.0
16	Phenol, 2,6-dimethoxy-4-(2-propenyl)-	S	1.1	1.1	1.5	1.2
17	Ethanone, 1-(4-hydroxy-3,5-dimethoxyphenyl)-	S	2.4	2.5	3.1	2.1
18	3,5-Dimethoxy-4-hydroxyphenylacet	S	1.9	0.0	0.0	0.0

	ic acid					
19	3,5-di-tert-Butyl-4-hydroxyphenylpropionic acid	H	0.0	0.0	0.0	0.0
20	Phenol, 2-methyl-	H	0.0	0.1	0.0	0.0
21	Phenol, 4-ethyl-	H	0.0	0.1	0.0	0.0
22	Phenol, 2-methoxy-4-(1-propenyl)-	G	0.0	0.3	0.0	0.0
23	Vanillin	G	0.0	0.4	0.0	0.5
24	Butylated Hydroxytoluene	H	0.0	29.0	14.8	5.4
25	Benzaldehyde, 4-hydroxy-3,5-dimethoxy-	S	0.0	1.3	1.5	1.9
26	2-Methoxy-6-methylphenol	G	0.0	0.0	0.7	0.0
27	Phenol, 2-methoxy-3-methyl-	G	0.0	0.0	0.0	0.5
28	1,2-Benzenediol, 3-methoxy-	G	0.0	0.0	0.0	9.5
	H%		5.4	29.3	15.2	6.1
	G%		73.9	53.1	60.5	72.3
	S%		20.5	18.7	24.4	21.7
	S/G		0.34	0.45	0.51	0.35

Table S2

Assignments of main lignin ^{13}C-^{1}H cross signals in the 2D HSQC spectra of the UL and L170.

Label	δ_C/δ_H	Assignment
**B_β**	**54.3/2.99**	**C_β−H_β in β-β (resinol) substructures (B)**
**−OCH_3**	**56.4/3.68**	**C−H in methoxyls**
**A_γ**	**60.4//3.30−3.79**	**C_γ−H_γ in β-O-4 substructures (A)**
**Est**	**62.7/4.30**	**γ-ester of LCC**
**Ara-6**	**63.3/3.40**	**C_6−H_6 in arabinosyl units**
**C_γ**	**63.3/3.66**	**C_γ−H_γ in phenylcoumaran substructures (C)**
**B_γ**	**71.8/3.75−4.12**	**C_γ−H_γ in β-β resinol substructures (B)**
**A_α**	**72.6/4.81**	**C_α−H_α in β-O-4 substructures (A)**
**Glu-5**	**73.3/3.25**	**C_5−H_5 in glucosyl units**
**$A_{\beta(G/H)}$**	**84.3/4.23**	**C_β−H_β in β-O-4 linked to G/H unit (A)**
**B_α**	**85.7/4.59**	**C_α−H_α in β-β resinol substructures (B)**
**$A_{\beta(S)}$**	**86.7/4.06**	**C_β−H_β in β-O-4 linked to a S unit (A)**
**C_α**	**87.7/5.38**	**C_α−H_α in phenylcoumaran substructures (C)**
**$S_{2,6}$**	**104.5/6.63**	**$C_{2,6}$−$H_{2,6}$ in syringyl units (S)**
**$S'_{2,6}$**	**107.2/7.26**	**$C_{2,6}$−$H_{2,6}$ in oxidized S units (S′)**
**G_2**	**111.8/6.94**	**C_2−H_2 in guaiacyl units (G)**
**G'_2**	**112.0/7.52**	**C_2−H_2 in oxidized G units (G′)**
**PCA_8**	**114.2/6.27**	**C_8−H_8 in *p*-coumaric acid (PCA)**
**$PCA_{3,5}$**	**115.2/6.71**	**$C_{3,5}$−$H_{3,5}$ in *p*-coumaric acid (PCA)**
**G_5**	**115.6/6.65**	**C_5−H_5 in guaiacyl units (G)**
**G_6**	**119.6/6.75**	**C_6−H_6 in guaiacyl units (G)**
**J_β**	**125.6/6.82**	**C_β-H_β in cinnamaldehyde end groups (J)**
**$H_{2,6}$**	**129.2/7.17**	**$C_{2,6}$−$H_{2,6}$ in H units (H)**
